# Therapy-related Acute Lymphoblastic Leukaemia has a Unique Genetic Profile Compared to *De Novo* Acute Lymphoblastic Leukaemia

**DOI:** 10.7150/jca.76719

**Published:** 2022-09-11

**Authors:** Hye Won Kook, Jin Ju Kim, Mi Ri Park, Ji Eun Jang, Yoo Hong Min, Seung-Tae Lee, Saeam Shin, June-Won Cheong

**Affiliations:** 1 Division of Hematology, Department of Internal Medicine, Yonsei University College of Medicine, Seoul 03722, Republic of Korea.; 2 Department of Laboratory Medicine, Yonsei University College of Medicine, Seoul 03722, Republic of Korea.

**Keywords:** next-generation sequencing, therapy-related acute lymphoblastic leukaemia, germline predisposition, *de novo* acute lymphoblastic leukaemia, mutation

## Abstract

**Background**: Unlike therapy-related myeloid neoplasms, therapy-related acute lymphoblastic leukaemia (tr-ALL) is poorly defined due to its rarity. However, increasing reports have demonstrated that tr-ALL is a distinct entity with adverse genetic features and clinical outcomes.

**Methods:** We compared the clinicopathological characteristics and outcomes of patients diagnosed with tr-ALL (*n* = 9) or *de novo* ALL (dn-ALL; *n* = 162) at a single institution from January 2012 to March 2021. The mutational landscapes of eight tr-ALL and 63 dn-ALL patients were compared from a comprehensive next-generation sequencing panel.

**Results**: All tr-ALL patients had the B-cell phenotype. The most frequently mutated genes were *IKZF1* (37%), *CDKN2A* (14%), *SETD2* (13%), and *CDKN2B* (11%) in dn-ALL, whereas *TP53* (38%) and *RB1* (25%) mutations were most common in tr-ALL. tr-ALL patients did not show a statistically significant difference in overall survival (*p* = 0.70) or progression-free survival (*p* = 0.94) compared to dn-ALL patients.

**Conclusions:** In this study, we determined the clinical and genetic profiles of Korean patients with tr-ALL. We found alterations in genes constituting the *TP53/RB1* pathway are more frequent in tr-ALL. Due to the rarity of the disease, multi-institutional studies involving a larger number of patients are required in future study.

## Introduction

Therapy-related leukaemia is defined as leukaemia arising because of the mutagenic effect of chemotherapy or radiotherapy. Its incidence is increasing with the development of effective cancer treatment options. Therapy-related myeloid neoplasms (t-MNs), which account for 10-20% of all cases of myeloid neoplasms, are classified by a distinguishable diagnosis 1,2. They include acute myeloid leukaemia, myelodysplastic syndromes, and myelodysplastic/myeloproliferative neoplasms, and their prognoses are poorer than those of *de novo* myeloid neoplasms, as evidenced by lower response rates to conventional therapies and inferior survival outcomes 2.

Therapy-related acute lymphoblastic leukaemia (tr-ALL), on the other hand, has not been categorized by the World Health Organization (WHO) due to its rarity. tr-ALL accounts for 3% to 9% of adult ALL 3. This entity has been recently recognized by clinicians and several studies, yet there is no fixed consensus on its definition. tr-ALL is sometimes grouped with secondary ALL, which is defined as ALL with a concomitant malignancy, regardless of prior treatment. However, several reports have demonstrated that tr-ALL is a distinct entity with adverse genetic features and clinical outcomes 4-7. It was demonstrated that tr-ALL imparts more severe outcomes compared to *de novo* ALL (dn-ALL), and some characteristic mutations of tr-ALL, including *KMT2A* (*MLL*) rearrangements, have been reported at a relatively high frequency 4.

In this study, we report the clinical characteristics, genetic abnormalities, and outcomes of patients diagnosed with tr-ALL at a single institution in Korea.

## Methods

### Patients and endpoints

We retrospectively reviewed all consecutive cases of adult patients (age ≥15 years) newly diagnosed with ALL at Severance Hospital, Yonsei University College of Medicine, between January 2012 and March 2021. This study was approved by the Institutional Review Board of Yonsei University College of Medicine and was conducted in accordance with the tenets of the Declaration of Helsinki (IRB No. 4-2021-0384). tr-ALL was defined as ALL occurring after chemotherapy or radiation exposure. Patients with ALL and a history of prior malignancy diagnosis but without exposure to cytotoxic therapy were classified as dn-ALL. Progression-free survival (PFS) was defined as the time from ALL diagnosis to the first relapse or death. Overall survival (OS) was measured from the date of confirmed diagnosis to the date of death for any reason or to the last follow-up.

### Sample processing, cytogenetic analysis, and molecular genetic analysis

Conventional G-banding karyotyping was performed using heparinized bone marrow aspirate following standard protocols. A complex karyotype was defined by the presence of three or more chromosomal abnormalities. Reverse-transcription polymerase chain reaction (RT-PCR) was performed using a HemaVision kit (DNA Technology, Aarhus, Denmark) targeting 28 recurrent translocations. Targeted NGS was performed with custom probes targeting 497 genes related to hematologic neoplasms (Table S1). After genomic DNA was extracted from diagnostic bone marrow aspirate, prepared libraries were hybridized with capture probes and sequenced using NextSeq 550Dx (Illumina, San Diego, CA, USA). Regarding the tr-ALL cases, germline matched analysis was performed using skin fibroblasts or bone marrow at complete remission to exclude the possibility of germline cancer-predisposing mutations. All procedures were performed according to the manufacturer's instructions. The Burrows-Wheeler alignment tool was used for sequence alignment. The sequencing reads were aligned to the NCBI human reference genome (hg19) using BWA (version 0.7.15), including coverage and quality assessment, single-nucleotide variant (SNV) and insertion/deletion (indel) detection, annotation, and prediction of deleterious mutational effects. Samtools and Pindel were used to analyze SNVs and indels. Variants with a variant allelic frequency (VAF) of 5% or higher were prioritized for further processing and annotation.

### Statistical analysis

Continuous variables were evaluated for normality using the Shapiro-Wilk test. In accordance with the distribution of independent variables, the Mann-Whitney U test or independent two-sample t-test was employed. The chi-squared test and Fisher's exact test were used to compare categorical variables. All parameters without normal distributions were presented as median with first and third quartiles. The OS and PFS were estimated using the Kaplan-Meier method. Cox proportional hazards regression was used to determine which factors had significant effects on survival and disease progression. All statistical analyses were performed using SPSS 25.0 software (IBM SPSS Statistics, Armonk, NY). *p* value < 0.05 was considered statistically significant.

## Results

### Comparison of clinical and pathologic characteristics of tr-ALL and dn-ALL

A total of 171 ALL patients was included. Among them, 9 (5.3%) were classified as tr-ALL. The clinical characteristics of the eligible patients are summarized in Table 1. Compared to dn-ALL patients, tr-ALL patients tended to be older at diagnosis (median 56 vs. 42 years, *p* = 0.06). All tr-ALL patients had the B-cell phenotype, while dn-ALL patients were diagnosed with various phenotypes, including B-cell (78.4%), T-cell (13.6%), mixed-phenotype acute leukaemia (MPAL) (6.8%), and Burkitt type (1.2%). The positive rate of *BCR-ABL1* rearrangement (Philadelphia, Ph) in tr-ALL patients was not statistically different with that in dn-ALL patients, 44.4% vs. 36.4% (*p* = 0.727).

### Mutational landscapes of dn-ALL and tr-ALL

To understand the landscape of somatic mutations in adult ALL patients, we reviewed the available NGS results of 71 ALL patients (63 dn-ALL and 8 tr-ALL patients). Targeted NGS testing has been implemented as a routine test in newly diagnosed ALL patients since March 2017 at our institution. Therefore, NGS data on ALL patients diagnosed after March 2017 were retrospectively reviewed (63 dn-ALL and 5 tr-ALL patients). In the case of tr-ALL, we additionally performed target NGS testing in cases diagnosed before March 2017 and with frozen bone marrow aspirate (3 tr-ALL patients). One tr-ALL patient (P170) could not proceed with the NGS testing due to the absence of residual diagnostic bone marrow aspirate. We confirmed that all patients had no other solid tumor involving bone marrow or concurrent hematologic malignancies at the time of bone marrow aspirate sampling for the NGS testing. Most of the dn-ALL patients had B-cell phenotype (B-lymphoblastic leukaemia, BLL) (54/63, 85.7%). Ph positivity was detected in 26 dn-BLL patients (26/54, 48.1%) and 4 tr-BLL patients (4/8, 50.0%).

In total, we detected 71 somatic SNVs and 86 copy number alterations in 45 genes that were suspected of potential pathogenic mutations. The number of detected mutations ranged from 0 to 8 in dn-ALL patients. More mutations in dn-ALL (median, 2.0; IQR, 0.2-3.0) than tr-ALL patients (median, 1.5; IQR, 1.0-2.0) were identified, but statistical significance was not achieved (*p* = 0.52). The median VAF of SNVs was 28.4% (IQR, 10.7-44.3) in all investigated ALL patients. Regarding the BLL cases, the median VAF of SNVs in therapy-related cases was 29.40% and in *de novo* cases was 16.80% (Figure S1, *p* = 0.45).

**Figure 1:**
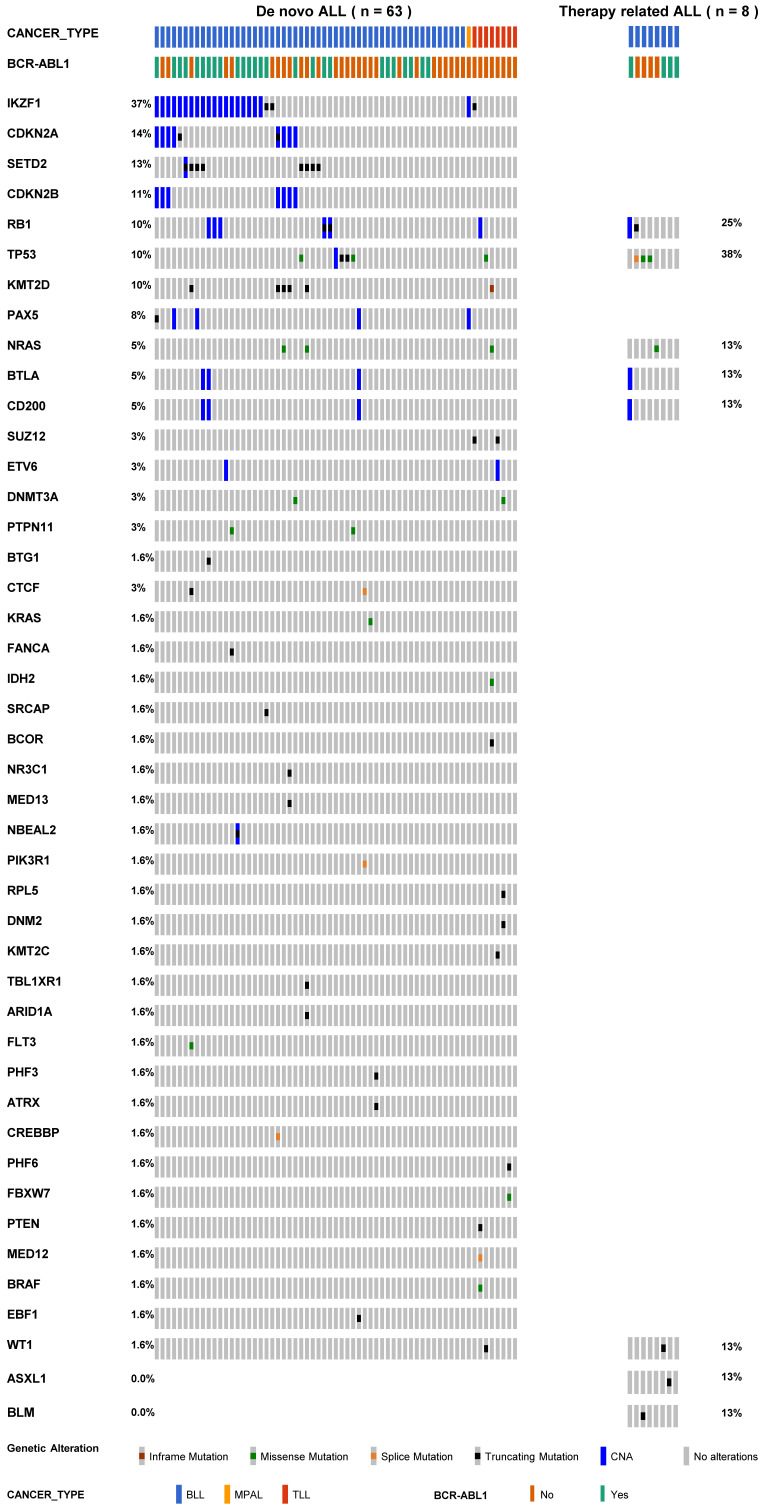
The mutational spectrum of 71 patients with acute lymphoblastic leukaemia.

The mutational profiles of 71 ALL patients are presented in Table 2 and Table S2. All tr-ALL mutations were verified as somatic through germline-match analysis. We observed obvious differences in terms of mutational landscape between dn- and tr-ALL patients. The most frequently mutated genes were *IKZF1* (37%), *CDKN2A* (14%), *SETD2* (13%), and *CDKN2B* (11%) in dn-ALL but *TP53* (38%) and *RB1* (25%) in tr-ALL (Figure 1). The mutations in *IKZF1* mostly coexisted in dn-BLL patients with *BCR-ABL1* translocation (15/21, 71.4%). However, *IKZF1* mutation was not found in tr-ALL patients with *BCR-ABL1* rearrangement. *TP53* and *RB1* mutations showed higher mutation frequencies in tr-ALL patients compared to dn-ALL patients, 38% vs*.* 10% and 25% vs*.* 10%, respectively. Even in patients with alterations in genes constituting the *TP53/RB1* pathway, including *CDKN2A/CDKN2B*, tr-ALL patients had a higher mutation frequency (50%, 4/8) than dn-ALL patients (33.3%, 21/63).

### Characteristics and treatment outcomes of the tr-ALL patients

The clinical and laboratory characteristics of the tr-ALL patients are shown in Table S3. The disease prior to tr-ALL onset was solid cancer in 8 patients and hematologic malignancy in one patient. Five patients were treated with systemic chemotherapy and operation as initial therapy for the prior diseases; 2 received systemic chemotherapy alone, 1 underwent operation, systemic chemotherapy, and local radiation; and 1 received trans-arterial chemoembolization (TACE) and radiofrequency ablation (RFA). The median duration from the time of prior disease diagnosis to the time of tr-ALL diagnosis was 6.4 years. Only one patient had residual lesions from their prior malignancy at the time of tr-ALL diagnosis.

Of the 8 patients who received systemic chemotherapy, 7 had available information regarding chemotherapy regimen. Among the chemotherapeutic agents used, anthracyclines were the most administered drug as part of prior therapy (*n* = 6), followed by alkylating agents (*n* = 5), antimicrotubules (taxanes) (*n* = 3), antimetabolites (*n* = 2), camptothecin analogues (*n* = 1), and retinoids (*n* = 1).

All but one tr-ALL patients received conventional induction therapy. The HyperCVAD regimen with or without tyrosine kinase inhibitors (TKIs) was most commonly used (*n* = 7) for induction therapy. One 27-year-old patient (P116) was treated with the pediatric GRAALL regimen, regarding his age. One patient (P27) was transferred from an outside hospital after achieving remission on the VPD regimen. Among TKIs, imatinib was administered to all Ph-positive patients (*n* = 4). After first-line treatment, 8 patients achieved complete remission (CR), and 1 failed to respond. The median time from diagnosis to the first CR was 1.1 months (0.9-1.6). Regarding post-remission treatment, 4 patients received allogeneic hematopoietic stem cell transplant (allo-HSCT). Among 9 tr-ALL patients, 4 are now alive in CR, including three of four patients who received allo-HSCT.

The median follow-up period for all patients was 58.3 months (IQR, 0.0-98.1). The median OS duration between the groups showed no statistical significance (12.7 months for tr-ALL and 32.6 months for dn-ALL patients, *p* = 0.70) as well as the median PFS (10.4 months for tr-ALL and 13.4 months for dn-ALL patients, *p* = 0.18) (Figure S2).

In the dn-ALL group, age at diagnosis [hazard ratio (HR), 1.03; 95% confidence interval (CI), 1.02-1.05; *p* < 0.001] and presence of myelodysplastic syndrome (MDS)-like cytogenetic abnormalities (HR, 6.26; 95% CI, 2.22-17.65; *p* = 0.001) were associated with poor survival in univariate analyses. In multivariate analysis of OS, only age at diagnosis (HR, 1.04; 95% CI, 1.02-1.06; *p* < 0.001) was associated with poor survival. Similarly, age at diagnosis (HR, 1.03; 95% CI, 1.02-1.04; *p* < 0.001) and MDS-like cytogenetic abnormalities (HR, 3.50; 95% CI, 1.32-9.25; *p* = 0.012) were associated with shorter PFS. In multivariate analysis of PFS, only age at diagnosis (HR, 1.03; 95% CI, 1.02-1.05; *p* < 0.001) was associated with shorter PFS. In the tr-ALL group, many factors, including age at diagnosis, CR achievement, HSCT, chromosomal abnormalities, prior cancer status at ALL diagnosis, time from prior malignancy diagnosis to ALL diagnosis, and topoisomerase II administration were analyzed as prognostic factors for survival and relapse, but statistical significance was not achieved.

## Discussion

Due to disease rarity, our knowledge regarding tr-ALL is limited. Unlike t-MNs, tr-ALL is not currently defined by the WHO as a sole disease entity. Some reports have analyzed tr-ALL regardless of how the primary malignancy was treated, 8,9 and other studies have focused on patients who received chemotherapy or radiotherapy 4,5. Focusing on the effects of chemotherapy and radiotherapy that might have caused genomic instability, our study investigated tr-ALL patients who underwent chemotherapy or radiotherapy.

Previous studies have reported that *KMT2A* rearrangement is a common abnormality in tr-ALL 4,6. *KMT2A* rearrangement is the prototypical cytogenetic finding among tr-AML patients exposed to topoisomerase II inhibitors, and the incidence of *KMT2A* rearrangement is higher in tr-ALL compared to dn-ALL 4,6. We studied one tr-ALL patient with *KMT2A-EPS15* rearrangement (P116). The patient had a history of alkylating therapy along with topoisomerase II inhibitor treatment. Future study with a larger number of tr-ALL patients is necessary to fully investigate the relationship between *KMT2A* rearrangement and tr-ALL.

In this study, we identified a total of 71 adult ALL cases with available genetic mutation information (assessed by NGS), including 54 dn-BLL and 8 tr-BLL patients who were previously diagnosed with a malignancy. A notable observation of our study is that *TP53* and *RB1* alterations were more frequent in tr-BLL than in dn-BLL patients. The role of *TP53* in development of t-MNs after exposure to topoisomerase II inhibitor and alkylating agent has been well described 1. Frequent *TP53* alteration, including copy number alteration in t-MN 10 and tr-BLL, has been reported 6. Somatic *TP53* alteration is suggested to influence defects in the DNA damage response in t-MN 10 as well as development of tr-BLL 6. *RB1*, a cell cycle regulator, is altered recurrently in t-MN 11 and ALL 12. There have been no previous reports demonstrating frequent *RB1* mutation in tr-ALL. However, higher frequency of *TP53/RB1* tumor suppressor pathway mutations in our tr-ALL cohort suggests overlapping features in high-risk genetic subtypes, as reported in high-risk BLL 13. However, the sample size is limited, and the results need to be verified.

Of all investigated tr-ALL patients, 33.3% had a family history of cancer, but only somatic mutations were found in our study. The possible involvement of cancer-predisposing genes not included in our target panel cannot be ruled out, but germline mutation was not found in common oncogenes, including *BRCA1, BRCA2, TP53*, *DDX41*, *RUNX1*, *ANKRD26*, and *ETV6*. Recent study reported a tr-ALL case in Li-Fraumeni syndrome patient 6. In a study by Churpek et al., the cancer susceptibility gene was screened in patients with tr-ALL among the breast cancer survivors, and *TP53* mutation was found in two of the four tr-ALL patients who underwent the test 5. Since very few studies have previously investigated germline presentation genes in tr-ALL patients, future study with large series is required for identifying the exact frequency of germline mutations among tr-ALL patients.

According to previous studies, tr-ALL has a poorer prognosis than dn-ALL 4,14. In our study, we could not demonstrate statistical significance between two groups. As previously demonstrated by other studies, tr-ALL patients were older than dn-ALL patients at diagnosis, and this might be explained by the 6.4-year median time from diagnosis of prior malignancy to ALL diagnosis in our study. This duration was similar to that reported by other groups 5,15.

Breast cancer was one of the most common prior malignancies in our study, in concordance with other reports, possibly due to its relatively superior prognosis to other cancers and frequent use of topoisomerase II inhibitors and alkylating agents for its treatment. Another common prior cancer was osteosarcoma. This might be due to the usage of doxorubicin in its treatment regimen, which is also a topoisomerase II inhibitor. In addition, most osteosarcomas occur in children or young adults aged 10 to 30 years.

The limitations of this study are mainly related to its retrospective nature and small sample size. Since data collection was performed from patients diagnosed in a period of 12 years, rapidly changing therapeutic options for prior malignancies and ALL might have caused bias. Also, although we conducted additional cytogenetic studies and mutational analysis with available samples, minimal residual disease data are lacking from the earlier patients. Multi-center studies with a sufficient number of patients using propensity score matching analysis are needed for statistically significant, minimally biased results. In this way, the emerging roles of bispecific antibodies and chimeric antigen receptor therapy in tr-ALL are promising directions for future study.

## Supplementary Material

Supplementary figures and tables.

## Figures and Tables

**Table 1:** Overall comparison of *de novo* ALL and therapy-related ALL^*^

Patient characteristics	All patients	*De novo* ALL	Therapy-related ALL	*p* value
Number	171	162	9	
Age at diagnosis, years	42 (28-56)	42 (27-56)	56 (49-61)	0.06
**Sex**				
Male	98 (57.3%)	93 (57.4%)	5 (55.6%)	0.913
Female	73 (42.7%)	69 (42.6%)	4 (44.4%)	
**Immunophenotype**				0.485
B	136 (79.5%)	127 (78.4%)	9 (100.0%)	
T	22 (12.9%)	22 (13.6%)	0 (0.0%)	
MPAL	11 (6.4%)	11 (6.8%)	0 (0.0%)	
Burkitt type	2 (1.2%)	2 (1.2%)	0 (0.0%)	
**Cytogenetics**				0.142
Normal	48 (28.1%)	45 (27.8%)	3 (33.3%)	0.712
*BCR-ABL1* rearrangement	63 (36.8%)	59 (36.4%)	4 (44.4%)	0.727
*KMT2A* rearrangement	6 (3.5%)	5 (3.1%)	1 (11.1%)	0.281
MDS-like^†^	5 (2.9%)	5 (3.1%)	0	1.000
Complex	6 (3.5%)	5 (3.1%)	1 (11.1%)	0.281
Other	43 (25.1%)	43 (26.5%)	0	0.114
PB NLR	0.46 (0.19-1.32)	0.44 (0.19-1.33)	0.51 (0.33-5.62)	0.510
PB blast (%)	49.0 (5.5-78.0)	44.7 (4.8-79.0)	24.0 (12.5-36.5)	0.373
PB WBC count (×10^9^/L)	23.21 (6.98-79.88)	67.69 (7.07-80.18)	9.43 (3.23-26.6)	0.348
BM blast (%)	85.0 (73.8-90.6)	85.0 (74.5-90.6)	88.8 (74.7-94.7)	0.993
LDH (IU/L)	665 (394-1407)	655 (394-1421)	824 (195-1861)	0.688

^*^Data are presented as median (interquartile ranges) or number (%); ^†^MDS-like cytogenetic abnormalities included deletions of chromosomes 5, 7, 11, 13, 17, and 20, as well as trisomy 8.

**Table 2:** Medical history and mutational spectra of 9 therapy-related ALL patients

Patient ID	Sex/age	Time from first cancer to ALL (years)	Prior malignancy	Prior cytotoxic agents	FHx	Mutation (VAF %)	*BCR-ABL1* rearrangement
P170	F/49	6.0	Breast cancer	Adriamycin, cyclophosphamide, paclitaxel	None	Not done	Negative
P168	M/64	10.3	Stomach cancer	5FU, adriamycin	None	*TP53* p.Val172Gly (34.2)	Negative
P116	M/21	1.5	Osteosarcoma	Ifosfamide, adriamycin, cisplatin	None	*KRAS* p.Gln61Leu (29.4) *NRAS* p.Gly12Asp (6.9)	Negative^*^
P104	F/64	5.2	Rectal cancer	Oxaliplatin, 5-FU	Lung cancer (brother)	*ASXL1* p.Gly646TrpfsTer12 (29.1)	Minor e1a2
P35	F/56	5.5	Ovarian cancer	Docetaxel, carboplatin, paclitaxel, liposomal doxorubicin, belotecan,	Pancreatic cancer (father)	*TP53* c.994-1G>A (90.7)	Negative
				cisplatin		*RB1* p.Trp563Ter (77.1)	
P27	M/62	11.7	HCC	Unknown chemotherapy^†^	None	*WT1* p.Asp367GlyfsTer19 (6.6)	Minor e1a2
P21	M/46	26.8	Osteosarcoma, AGC	Unknown chemotherapy	None	*TP53* p.Arg248Gln (49.5)	Negative
						*BLM* p.Ile893GlufsTer70 (11.2)	
P4	M/52	6.4	APL	ATRA, idarubicin	Colon cancer (father), gastric cancer (brother)	Not detected	Minor e1a2
P1	F/59	13.7	Breast cancer, Thyroid cancer	Adriamycin, cyclophosphamide, paclitaxel	None	*BTLA* whole gene deletion	Minor e1a2
						*CD200* exon 2-7 deletion	
						*RB1* exon 18-27 deletion	

^*^KMT2A-EPS15 rearrangement-positive; ^†^Trans-arterial chemoembolization.

## Abbreviations

tr-ALL: therapy-related acute lymphoblastic leukaemia
WHO: World Health Organization
dn-ALL: de novo acute lymphoblastic leukaemia
t-MNs: therapy-related myeloid neoplasms
PFS: progression-free survival
OS: overall survival
RT-PCR: reverse-transcription polymerase chain reaction
SNV: single-nucleotide variant
indel: insertion/deletion
VAF: variant allelic frequency
IQR: interquartile ranges
MPAL: mixed-phenotype acute leukaemia
Ph: Philadelphia
LDH: lactate dehydrogenase
BLL: B-lymphoblastic leukaemia
TACE: trans-arterial chemoembolization
RFA: radiofrequency ablation
hyper-CVAD: hyperfractionated cyclophosphamide, vincristine, adriamycin, and dexamethasone
TKIs: tyrosine kinase inhibitors
GRAALL: Group for Research on Adult Acute Lymphoblastic Leukaemia
VPD: vincristine, prednisone, and doxorubicin
CR: complete remission
allo-HSCT: allogeneic hematopoietic stem cell transplant
HR: hazard ratio
CI: confidence interval
MDS: myelodysplastic syndrome
CNA: copy number alteration
TLL: T-lymphoblastic leukaemia
PB: peripheral blood
NLR: neutrophil-lymphocyte ratio
FHx: family history of cancer in first-degree relatives
NT: nucleotide
AA: amino acid
HCC: hepatocellular carcinoma
AGC: advanced gastric cancer
APL: acute promyelocytic leukaemia
ATRA: all trans retinoic acid
TRUNC: truncation variant
SPLICE: splicing variant
NED: no evidence of disease
CR: complete remission
