# Supplementary material for: Therapy-related Acute Lymphoblastic Leukaemia has a Unique Genetic Profile Compared to De Novo Acute Lymphoblastic Leukaemia
Source: J Cancer. 2022 Sep 11;13(12):3326–32. doi: 10.7150/jca.76719 (PMC9516015; doi:10.7150/jca.76719)

Table S1. List of 497 genes included in the targeted gene panel

ABCB1, ABCB7, ABCG2, ABCG5, ABCG8, ABL1, ABL2, ACD, ACTB, ACTN1, ADA, ADAMTS13, AIRE, AK1, AK2, AKT2, ALAS2, ALDOA, AMN, ANK1, ANKRD26, AP3B1, ARID1A, ARPC1B, ASXL1, ATG2B, ATM, ATR, ATRX, AXIN1, BCL11B, BCL2, BCL6, BCOR, BCORL1, BHLHE41, BIRC3, BLM, BPGM, BRAF, BRCA1, BRCA2, BRCC3, BRINP3, BRIP1, BTG1, BTK, BTLA, C3, C4BPA, C4BPB, CALN1, CALR, CARD11, CASP10, CBL, CBLB, CBLC, CCND1, CD200, CD247, CD27, CD36, CD3D, CD3E, CD40LG, CD46, CD58, CD59, CD79B, CDAN1, CDKN1B, CDKN2A, CDKN2B, CEBPA, CFB, CFH, CFHR1, CFHR3, CFHR4, CFHR5, CFI, CHD1, CHD4, CHD9, CHMP2B, CLPB, CNOT3, COX4I2, CREBBP, CRLF2, CSF1R, CSF2RA, CSF3R, CTC1, CTCF, CTSC, CUBN, CUX1, CXCR4, CYB5R3, CYBA, CYBB, CYCS, DCLRE1C, DDX41, DGKE, DGKH, DHFR, DIS3, DKC1, DNM2, DNMT1, DNMT3A, EBF1, ECT2L, EED, EGFR, EGLN1, EGLN2, EGLN3, EHMT1, ELANE, EP300, EPAS1, EPB41, EPB42, EPCAM, EPO, EPOR, ERCC4, ERG, ETNK1, ETV6, EZH2, F2R, FANCA, FANCB, FANCC, FANCD2, FANCE, FANCF, FANCG, FANCI, FANCL, FANCM, FAS, FASLG, FAT1, FBXW7, FCGR1A, FCGR3B, FERMT3, FLI1, FLNA, FLT3, FOXP3, G6PC3, G6PD, GATA1, GATA2, GATA3, GCLC, GFI1, GFI1B, GIF, GINS1, GLRX5, GNAS, GNB1, GP1BA, GP1BB, GP9, GPI, GPRC5A, GPX1, GSKIP, GSN, GSR, GSS, HAX1, HBA1, HBA2, HBB, HBD, HCLS1, HFE, HIF1A, HIF1AN, HIF3A, HK1, HNRNPK, HOOK1, HOXA10, HOXA11, HRAS, HSPA9, HUWE1, ID3, IDH1, IDH2, IFNG, IFNGR1, IFNGR2, IKZF1, IKZF2, IKZF3, IL12RB1, IL2RB, IL2RG, IL3RA, IL7R, IRF1, ITGA2, ITGA2B, ITGB2, ITGB3, ITK, ITPKB, JAGN1, JAK1, JAK2, JAK3, JAKMIP2, JMJD1C, KDM5C, KDM6A, KDM7A, KIF23, KIT, KLF1, KMT2A, KMT2C, KMT2D, KRAS, LAMB4, LAMTOR2, LAPTM5, LCK, LEF1, LIG4, LMNA, LMO1, LMO2, LPIN2, LRP1B, LRRC4, LUC7L2, LYL1, LYST, MAD2L2,

MAGT1, MAP2K1, MAP2K2, MASTL, MBL2, MECOM, MED13, MEF2B, MEF2C, MEFV, MET, MLH1, MLLT10, MLLT3, MPL, MSH2, MSH4, MSH6, MTA1, MTAP, MTR, MTRR, MVK, MYB, MYC, MYD88, MYH9, MYSM1, NAF1, NBEAL2, NBN, NCF2, NCOR2, NF1, NHEJ1, NHP2, NLRP3, NOD2, NOP10, NOTCH1, NOTCH2, NPM1, NR3C1, NRAS, NT5C2, NT5C3A, NTRK3, NUP214, OS9, P2RY2, PALB2, PARN, PAX5, PBX1, PC, PCDHB1, PDGFRA, PDGFRB, PDHA1, PDHX, PFKL, PFKM, PGK1, PGM3, PHF6, PICALM, PIEZO1, PIGA, PIK3CD, PIK3R1, PKLR, PML, PMS2, PNP, POT1, PRDM1, PRF1, PRKACG, PRPF40B, PTCH2, PTEN, PTK2B, PTPN11, PTPN2, PTPRC, PTPRD, PUS1, RAB27A, RAC1, RAC2, RAD21, RAD50, RAD51, RAD51C, RAF1, RAG1, RAG2, RB1, RBBP6, RBM8A, RELN, RFWD3, RHAG, RHOA, RIT1, RMRP, RNF168, RPL10, RPL11, RPL15, RPL23, RPL26, RPL27, RPL31, RPL35A, RPL36, RPL5, RPS10, RPS14, RPS15, RPS17, RPS19, RPS24, RPS26, RPS27, RPS27A, RPS28, RPS29, RPS7, RTEL1, RUNX1, RUNX1T1, SAMD9L, SBDS, SBF2, SEC23B, SERPING1, SETBP1, SETD2, SF1, SF3A1, SF3B1, SH2B3, SH2D1A, SHOC2, SLC11A2, SLC19A2, SLC25A38, SLC2A1, SLC35C1, SLC37A4, SLC4A1, SLCO1B1, SLCO1B3, SLFN14, SLX4, SMARCD2, SMC1A, SMC3, SOS1, SPINK5, SPRED1, SPTA1, SPTB, SRC, SRCAP, SRP72, SRSF2, STAG1, STAG2, STAT3, STAT5B, STEAP3, STX11, STXBP2, SUZ12, SYNE1, TAL1, TAL2, TAZ, TBL1XR1, TBX1, TCF3, TCIRG1, TEC, TERC, TERF1, TERF2, TERF2IP, TERT, TET1, TET2, TET3, THBD, THPO, TINF2, TLX1, TLX3, TMPRSS6, TNFAIP3, TNFRSF13B, TNFRSF14, TNFRSF1A, TOX, TP53, TPI1, TPMT, TRAF3, TRNT1, TSLP, TSR2, TUBB1, TYK2, U2AF1, U2AF2, UBE2T, UGT1A1, UGT1A7, UNC13B, UNC13D, UNC5D, USB1, USH2A, USP9X, VHL, VPS13B, VPS45, VWF, WAS, WDR1, WIPF1, WRAP53, WT1, XBP1, XIAP, XK, XRCC2, YARS2, ZAP70, ZFHX4, ZNF197, ZRSR2, MRE11A, WHSC1, STON1, OBFC1

Table S2. The mutational profiles of 63 *de novo* acute lymphoblastic leukaemia patients

Patient ID	Diagnosis	Gene	Transcript number	NT alteration	AA alteration	Type	VAF (%)
P2	BLL	*IKZF1*	NM_006060.4	Partial gene deletion		CNA	
		RB1	NM_000321.2	Partial gene deletion		CNA	
P3	BLL	*IKZF1*	NM_006060.4	Partial gene deletion		CNA	
		CD200	NM_001004196.3	Whole gene deletion		CNA	
		BTLA	NM_181780	Whole gene deletion		CNA	
		BTG1	NM_001731	c.295_325delinsTGAGG	p.Pro99Ter	TRUNC	8.1
		RB1	NM_000321.2	Partial gene deletion		CNA	
P5	BLL	*KRAS*	NM_033360.2	c.183A>C	p.Gln61His	MISSENSE	10.5
P6	BLL	*CDKN2A*	NM_000077	Whole gene deletion		CNA	
		CDKN2B	NM_004936	Partial gene deletion		CNA	
		IKZF1	NM_006060.4	Partial gene deletion		CNA	
P7	BLL	*IKZF1*	NM_006060.4	Partial gene deletion		CNA	
		FANCA	NM_000135.2	c.3720_3724del	p.Glu1240AspfsTer36	TRUNC	37.9
		PTPN11	NM_002834.4	c.1459G>A	p.Asp487Asn	MISSENSE	39.2
P8	BLL	*PTPN11*	NM_002834.4	c.182A>T	p.Asp61Val	MISSENSE	8.6
		TP53	NM_000546.5	c.536A>G	p.His179Arg	MISSENSE	84.3
P9	BLL	*CDKN2A*	NM_000077	Whole gene deletion		CNA	
		CDKN2B	NM_004936	Whole gene deletion		CNA	
		IKZF1	NM_006060.4	Partial gene deletion		CNA	
		PAX5	NM_016734.2	c.169C>T	p.Gln57Ter	TRUNC	32.9
P10	BLL	Not detected					
P11	TLL	*IDH2*	NM_002168.3	c.419G>A	p.Arg140Gln	MISSENSE	45.1
		NRAS	NM_002524.4	c.34G>C	p.Gly12Arg	MISSENSE	45.3

		KMT2D	NM_003482.3	c.11779_11793delinsAGGCAA	p.Gln3927_Leu3931delinsArgGln	INFRAME	26.9
		BCOR	NM_001123383.1	c.3163A>T	p.Lys1055Ter	TRUNC	93.7
P12	BLL	*IKZF1*	NM_006060.4	Partial gene deletion		CNA	
P13	BLL	Not detected					
P14	BLL	*IKZF1*	NM_006060.4	Partial gene deletion		CNA	
		SETD2	NM_014159.6	Partial gene deletion		CNA	
		SETD2	NM_014159.6	c.7509_7519delinsGGCGAA	p.Asp2504AlafsTer14	TRUNC	10.9
P15	BLL	*SETD2*	NM_014159.6	c.607_608del	p.Ser203IlefsTer33	TRUNC	8
P16	BLL	Not detected					
P17	BLL	*IKZF1*	NM_006060.4	Partial gene deletion		CNA	
		RB1	NM_000321.2	Partial gene deletion		CNA	
P18	BLL	*IKZF1*	NM_006060.4	Whole gene deletion		CNA	
P19	BLL	*TP53*	NM_000546.5	c.818G>A	p.Arg273His	MISSENSE	21.1
		TP53	NM_000546.5	c.846dup	p.Arg283AlafsTer23	TRUNC	8.3
P22	BLL	*IKZF1*	NM_006060.4	Partial gene deletion		CNA	
		PAX5	NM_016734.2	Partial gene deletion		CNA	
		SETD2	NM_014159.6	c.6626_6642delinsCAGGTCG	p.Leu2209Serfs*36	TRUNC	15.7
		SETD2	NM_014159.6	c.7575_7582del	p.Asn2526GlyfsTer6	TRUNC	6.9
P23	BLL	Not detected					
P24	BLL	Not detected					
P25	TLL	Not detected					
P26	TLL	*IKZF1*	NM_006060.4	c.908_909insT	p.Glu304ArgfsTer185	TRUNC	5.6
		SUZ12	NM_015355.3	c.856C>T	p.Arg286Ter	TRUNC	32.1
P28	BLL	*IKZF1*	NM_006060.4	Partial gene deletion		CNA	
		ETV6	NM_001987	Partial gene deletion		CNA	
P31	BLL	*CDKN2A*	NM_000077	Whole gene deletion		CNA	
		CDKN2B	NM_004936	Partial gene deletion		CNA	

		IKZF1	NM_006060.4	Partial gene deletion		CNA	
P32	BLL	*RB1*	NM_000321.2	c.1775_1776insGA	p.Asn593IlefsTer19	TRUNC	66.7
		RB1	NM_000321.2	Whole gene deletion		CNA	
P34	BLL	Not detected					
P36	MPAL	*IKZF1*	NM_006060.4	Partial gene deletion		CNA	
		PAX5	NM_016734.2	Partial gene deletion		CNA	
P37	BLL	*IKZF1*	NM_006060.4	c.1062_1069delinsACCTCCCCC	p.Ala355Profs*134	TRUNC	12.8
		SRCAP	NM_006662.2	c.4316_4317insG	p.Ile1441HisfsTer407	TRUNC	6.3
P38	BLL	*TP53*	NM_000546.5	Whole gene deletion		CNA	
P40	BLL	*SETD2*	NM_014159.6	c.5463_5467delinsCCACCC	p.Pro1822Hisfs*10	TRUNC	11.9
P41	BLL	*KMT2D*	NM_003482.3	c.12661C>T	p.Gln4221Ter	TRUNC	31
		CDKN2A	NM_000077	Whole gene deletion		CNA	
		CDKN2B	NM_004936	Whole gene deletion		CNA	
		NR3C1	NM_001018077.1	c.1275_1278dup	p.Glu427Ter	TRUNC	6.2
		MED13	NM_005121.2	c.4075dup	p.Met1359AsnfsTer21	TRUNC	32.1
P42	BLL	*TP53*	NM_000546.5	c.846dup	p.Arg283AlafsTer23	TRUNC	82.6
P43	BLL	*IKZF1*	NM_006060.4	Partial gene deletion		CNA	
		NBEAL2	NM_015175.2	Partial gene deletion		CNA	
		NBEAL2	NM_015175.2	c.3700del	p.Ala1234GlnfsTer4	TRUNC	
P44	BLL	*IKZF1*	NM_006060.4	Partial gene deletion		CNA	
P45	BLL	*PIK3R1*	NM_181523.2	c.1425+1G>C		SPLICE	15.8
		CTCF	NM_006565.3	c.1207+2T>C		SPLICE	28
P46	TLL	*RPL5*	NM_000969.3	c.357T>A	p.Tyr119Ter	TRUNC	28.8
		DNMT3A	NM_022552.4	c.2645G>A	p.Arg882His	MISSENSE	39.5
		DNM2	NM_001005360.2	c.1136_1137insAGGTGAGC	p.Phe379LeufsTer38	TRUNC	10.4
P47	BLL	*IKZF1*	NM_006060.4	Partial gene deletion		CNA	
P48	BLL	*SETD2*	NM_014159.6	c.5137_5138insC	p.Asp1713AlafsTer15	TRUNC	32.4
		SETD2	NM_014159.6	c.2837_2865delinsTGGG	p.Arg946MetTer28	TRUNC	6.1
		IKZF1	NM_006060.4	Whole gene deletion		CNA	

		CD200	NM_001004196.3	Whole gene deletion		CNA	
		BTLA	NM_181780	Whole gene deletion		CNA	
P49	BLL	Not detected					
P50	BLL	Not detected					
P51	BLL	Not detected					
P52	TLL	*SUZ12*	NM_015355.3	c.1150_1151del	p.Leu385ProfsTer10	TRUNC	42.1
		KMT2C	NM_170606.2	c.2104_2105dup	p.Glu704MetfsTer15	TRUNC	45.1
		ETV6	NM_001987	Whole gene deletion		CNA	
P53	BLL	*TBL1XR1*	NM_024665.5	c.297_298insTT	p.Arg100LeufsTer43	TRUNC	15.8
		SETD2	NM_014159.6	c.4187dup	p.Asn1396LysfsTer2	TRUNC	49.2
		NRAS	NM_002524.4	c.35G>A	p.Gly12Asp	MISSENSE	18.2
		KMT2D	NM_003482.3	c.11674C>T	p.Gln3892Ter	TRUNC	46.5
		ARID1A	NM_006015.4	c.1882_1883insAAAAG	p.Met628LysfsTer3	TRUNC	8.9
P54	BLL	*CDKN2A*	NM_000077	Partial deletion		CNA	
		CDKN2B	NM_004936	Partial deletion		CNA	
		NRAS	NM_002524.4	c.38G>T	p.Gly13Val	MISSENSE	35.5
		KMT2D	NM_003482.3	c.12844C>T	p.Arg4282Ter	TRUNC	41.2
P55	BLL	*SETD2*	NM_014159.6	c.6118C>T	p.Arg2040Ter	TRUNC	5.2
		KMT2D	NM_003482.3	c.8053C>T	p.Arg2685Ter	TRUNC	16.8
		KMT2D	NM_003482.3	c.7228C>T	p.Arg2410Ter	TRUNC	6.6
		KMT2D	NM_003482.3	c.6343_6346del	p.Ser2115ArgfsTer28	TRUNC	9
		KMT2D	NM_003482.3	c.2817_2818insAGGC	p.Ser940ArgfsTer30	TRUNC	5.4
		IKZF1	NM_006060.4	Partial deletion		CNA	
		FLT3	NM_004119.2	c.2503G>T	p.Asp835Tyr	MISSENSE	37.7
		CTCF	NM_006565.3	c.1176C>G	p.Tyr392Ter	TRUNC	6.3
P56	BLL	*PHF3*	NM_001290259.1	c.2728A>T	p.Lys910Ter	TRUNC	16.3
		ATRX	NM_000489.3	c.3064C>T	p.Arg1022Ter	TRUNC	49
P57	BLL	Not detected					
P59	BLL	*KMT2D*	NM_003482.3	c.5064_5065insGCCCCCA	p.Arg1689AlafsTer4	TRUNC	15.7

		CREBBP	NM_004380.2	c.3982+2_3982+13delTAAGTTTCGGGA		SPLICE	9.8
		CDKN2B	NM_004936	Whole gene deletion		CNA	
		CDKN2A	NM_000077	Whole gene deletion		CNA	
		CDKN2A	NM_000077	c.262G>T	p.Glu88Ter	TRUNC	80.5
P60	BLL	Not detected					
P62	BLL	Not detected					
P63	BLL	*IKZF1*	NM_006060.4	Partial gene deletion		CNA	
		CDKN2A	NM_000077	Whole gene deletion		CNA	
		PAX5	NM_016734.2	Whole gene deletion		CNA	
P64	TLL	*PHF6*	NM_001015877.1	c.297T>A	p.Cys99Ter	TRUNC	90.9
		FBXW7	NM_033632.3	c.1394G>T	p.Arg465Leu	MISSENSE	43.5
P65	BLL	*IKZF1*	NM_006060.4	c.1509C>G	p.Tyr503Ter	TRUNC	15.6
P67	TLL	*RB1*	NM_000321.2	Whole gene deletion		CNA	
		PTEN	NM_000314.4	c.698_701delinsGGGGGAGA	p.Arg234GlyfsTer10	TRUNC	49.6
		PTEN	NM_000314.4	c.734_740del	p.Gln245HisfsTer9	TRUNC	15.1
		MED12	NM_005120.2	c.205-2A>G		SPLICE	88.5
		BRAF	NM_004333.4	c.1780G>A	p.Asp594Asn	MISSENSE	45.1
P68	BLL	*PAX5*	NM_016734.2	Partial gene deletion		CNA	
		EBF1	NM_024007.3	c.1708_1714dupCCCACCT	p.Cys572SerfsTer28	TRUNC	29.5
		CD200	NM_001004196.3	Whole gene deletion		CNA	
		BTLA	NM_181780	Whole gene deletion		CNA	
P71	BLL	*TP53*	NM_000546.5	c.518T>C	p.Val173Ala	MISSENSE	45.1
		SETD2	NM_014159.6	c.7531_7532insG	p.Lys2511ArgfsTer9	TRUNC	20.6
P72	BLL	Not detected					
P73	BLL	*RB1*	NM_000321.2	c.1848dupA	p.Gly617ArgfsTer36	TRUNC	57.4
		RB1	NM_000321.2	Partial gene deletion		CNA	
P75	TLL	*WT1*	NM_024426.4	c.1372C>T	p.Arg458Ter	TRUNC	40
		TP53	NM_000546.5	c.560-27_560delinsTAGA	p.Gly187Asp	MISSENSE	23.7
		TP53	NM_000546.5	c.844C>T	p.Arg282Trp	MISSENSE	42.7

P76	BLL	*DNMT3A*	NM_022552.4	c.2644C>A	p.Arg882Ser	MISSENSE	46.7
		CDKN2B	NM_004936	Whole gene deletion		CNA	
		CDKN2A	NM_000077	Whole gene deletion		CNA	
P83	BLL	*IKZF1*	NM_006060.4	Whole gene deletion		CNA	
		CDKN2A	NM_000077	c.199_200insCTCG	p.Gly67AlafsTer54	TRUNC	19.8
P89	BLL	Not detected					
P155	BLL	Not detected					

Table S3. Characteristics and treatment outcomes of the tr-ALL patients (*n*=9)

Patient characteristics	Median (IQR) or number (%)
Duration from prior malignancy diagnosis to tr-ALL diagnosis, yrs (range)	6.4 (5.5-11.7)
Type of prior malignancy	
Solid cancer	8 (89)
Hematologic malignancy	1 (11)
Prior malignancy	
More than one prior diagnosis	2 (22)
Breast cancer	2 (22)
Stomach cancer	2 (22)
Osteosarcoma	2 (22)
Rectal cancer	1 (11)
Hepatocellular carcinoma	1 (11)
Ovarian cancer	1 (11)
Acute promyelocytic leukaemia	1 (11)
Thyroid cancer	1 (11)
Type of prior therapy	
Chemotherapy +/- Operation	7 (78)
Chemotherapy + Radiation +/- Operation	1 (11)
TACE and RFA	1 (11)
Chemotherapeutic agents used for prior malignancy	
Anthracyclines	6 (86)
Alkylating agents	5 (71)
Antimicrotubules (Taxanes)	3 (17)
Antimetabolites	2 (29)
Camptothecin analogues	1 (14)
Retinoids	1 (14)
Original disease status at tr-ALL diagnosis	
NED or CR	8 (89)
Residual or progression	1 (11)

ALL induction regimen	
Hyper-CVAD +/- TKIs	7 (78)
GRAALL	1 (11)
VPD	1 (11)
CR1 achievement after ALL induction	8 (89)
Allogenic hematopoietic stem cell transplantation	4 (44)
Matched sibling donor	2 (22)
Haploidentical donor	2 (22)

Figure S1. The variant allele frequencies (VAFs) of single-nucleotide variants from 63 *de novo* acute lymphoblastic leukaemia (dn-ALL), 54 *de novo* B-lymphoblastic leukaemia (dn-BLL), and 8 therapy-related BLL (tr-BLL) patients. The median VAFs of each group are 28.0% (IQR, 10.6-44.7), 16.80% (IQR, 8.93-37.85), and 29.40% (IQR, 10.13-56.4), respectively.

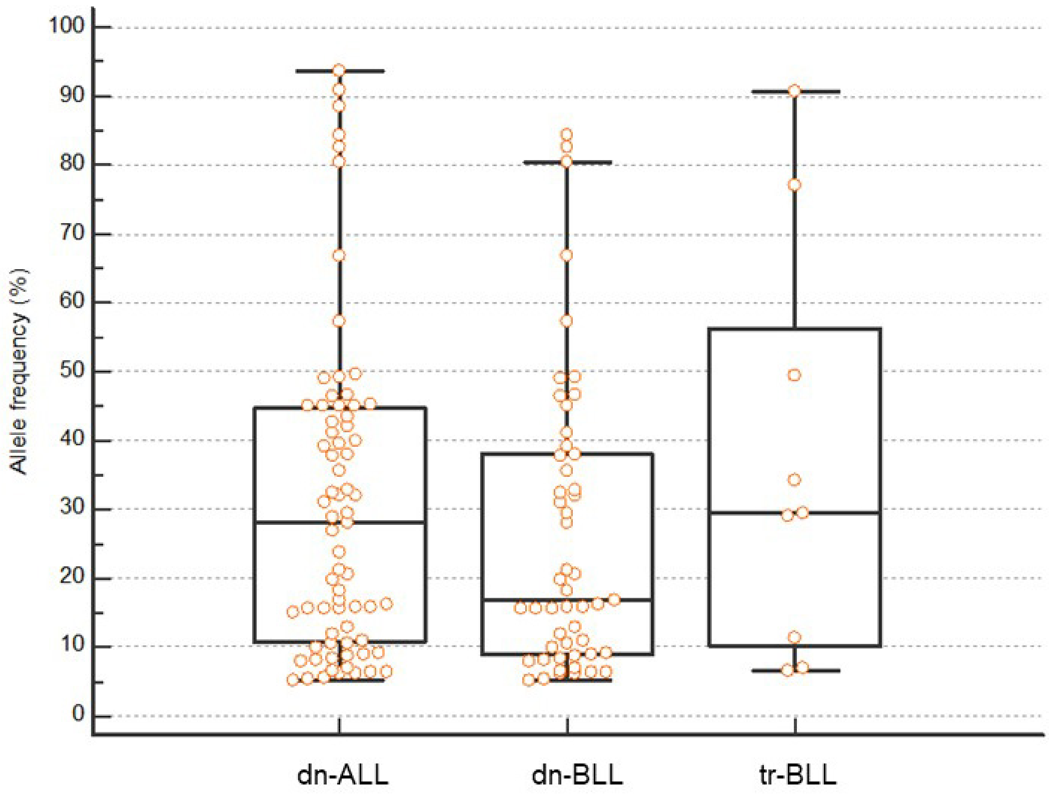

Figure S2. Survival outcome of *de novo* acute lymphoblastic leukemia (dn-ALL) and therapy-related ALL (tr-ALL). The differences in median (**A**) overall survival and (**B**) progression-free survival between dn-ALL and tr-ALL patients.

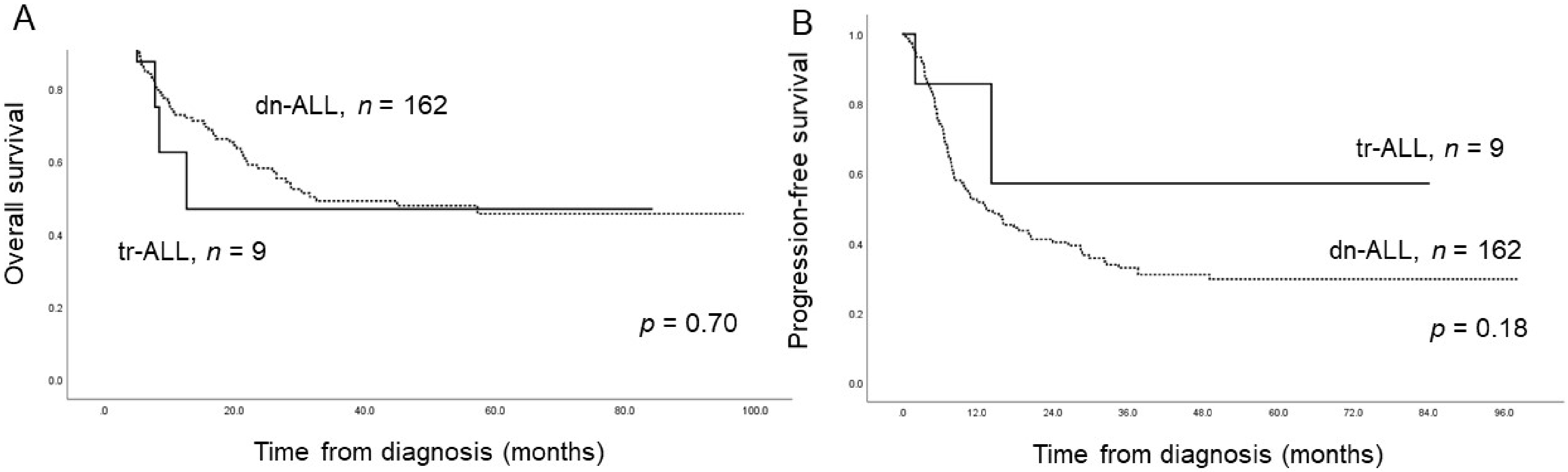

Supplement: Supplementary file 1 — Supplementary figures and tables. [file jcav13p3326s1.pdf]
